# Publisher Correction: Spectroelectrochemical study of water oxidation on nickel and iron oxyhydroxide electrocatalysts

**DOI:** 10.1038/s41467-020-14297-x

**Published:** 2020-01-16

**Authors:** Laia Francàs, Sacha Corby, Shababa Selim, Dongho Lee, Camilo A. Mesa, Robert Godin, Ernest Pastor, Ifan E. L. Stephens, Kyoung-Shin Choi, James R. Durrant

**Affiliations:** 1 Department of Chemistry, Imperial College London, White City Campus, London, W12 0BZ UK; 2 Department of Chemistry, University of Wisconsin-Madison, Madison, WI 53706 USA; 3 Department of Materials, Imperial College London, South Kensington Campus, London, SW7 2 AZ UK; 4 Present Address: The University of British Columbia, Department of Chemistry, 3247 University Way, Kelowna, V1V 1V7 BC Canada

**Keywords:** Catalytic mechanisms, Optical spectroscopy, Electrocatalysis

Correction to: *Nature Communications* 10.1038/s41467-019-13061-0, published online 15 November 2019.

The original version of this Article contained an error in Fig. 1, in which the vertical axis label incorrectly read ‘*J* (mA/cm^−2^)’ instead of ‘*J* (mA cm^−2^)’.

In addition, the seventh sentence in the legend of Fig. 1, originally, incorrectly read ‘Arrows indicate the onset of the catalysis, which has been approximated to the potential at which the current reaches 20 μA/cm^−2^.’ The correct version states ‘20 μA cm^−2^’ in place of ‘20 μA/cm^−2^’.

In the original version of Table 1, the second column for Entries 5, 8 and 9 incorrectly read ‘Overpotential@2.5 mA/cm^−2^ (V)’, ‘*τ* (s)@1 mA/cm^−2^’ and ‘TOF (s−1)@1 mA/cm^−2^’ respectively. The correct version states ‘mA cm^−2^’ instead of ‘mA/cm^−2^’ in each case.

The footnote to Table 1, originally, incorrectly read ‘^a^Catalysis onset approximated to the potential at which the current reaches 20 μA/cm^−2^’. The correct version states ‘20 μA cm^−2^’ in place of ‘20 μA/cm^−2^’.

In Fig. 2c, the original right vertical axis label incorrectly read ‘nmol of MOOH(+) or (++)/cm^−2^’ instead of ‘nmol of MOOH(+) or (++) cm^−2^)’.

In Fig. 2d, the original vertical axis label incorrectly read ‘Accumulated charge ([++]/cm^−2^)’ instead of ‘Accumulated charge ([++] cm^−2^)’.

The third and fourth sentences in the legend of Fig. 2, originally, incorrectly read ‘Normalised ΔO.D. spectra for the (+/++) oxidation wave correlated with water oxidation for FeOOH (orange), FeOOHNiOOH (green) and Ni(Fe)OOH (grey), obtained by subtracting spectra at OCP after activation from the spectra at 1 mA/cm^−2^. Concentrations of oxidised MOOH(+) states generated in the activation process (black) and of MOOH(++) states generated at a current density of 1 mA/cm^2^.’ The correct version replaces both occurrences of ‘1 mA/cm^2^’ with ‘1 mA cm^−2^’.

The fifth sentence of the second paragraph in the Spectroelectrochemical characterisation section of the Results and discussion incorrectly read ‘Moreover, at current densities equal to 1 mA/cm^2^,…’ The correct version states ‘1 mA cm^2^’ in place of ‘1 mA/cm^2^’.

In Fig. 4b, the original right vertical axis label incorrectly read ‘*J* (mA/cm^−2^)’ instead of ‘*J* (mA cm^−2^)’.

The fifth sentence of the second paragraph of the Kinetics of water oxidation section of the Results and discussion incorrectly stated ‘The catalytic TOFs (s^−1^) at 1 mA/cm^−2^ current density are indicated in Table 1, entry 9.’ The correct version states ‘1 mA cm^2^’ in place of ‘1 mA/cm^2^’.

The second sentence of the first paragraph of the Mechanism discussion section of the Results and discussion incorrectly stated ‘From the gradient of the plots presented in Fig. 5a, we find that all three electrocatalysts exhibit a reaction order (α) of approximately four under conditions of catalytic water oxidation (20 μA/cm^−2^–1 mA/cm^−2^).’ The correct version states ‘(20 μA cm^−2^–1 mA cm^−2^)’ in place of ‘(20 μA/cm^−2^–1 mA/cm^−2^)’.

The second sentence of the first paragraph of the Spectroelectrochemical experiments section of the Methods incorrectly stated ‘The measured data is generally presented as spectroelectrochemical difference spectra (ΔO.D.), which are generated by subtracting a reference spectrum (usually at the OCP or water-oxidation onset) from the absorption data obtained under conditions of interest (e.g., at current densities of 1 mA/cm^−2^, as in Fig. 2b).’ The correct version states ‘1 mA cm^2^’ in place of ‘1 mA/cm^2^’.

This has been corrected in both the PDF and the HTML versions of the Article.

