# Supplementary material for: Spectroelectrochemical study of water oxidation on nickel and iron oxyhydroxide electrocatalysts
Source: Nat Commun. 2019 Nov 15;10:5208. doi: 10.1038/s41467-019-13061-0 (PMC6858349; doi:10.1038/s41467-019-13061-0)
Supplement: Supplementary file 1 — Supplementary Information [file 41467_2019_13061_MOESM1_ESM.pdf]

Supplementary Information

Spectroelectrochemical study of water oxidation on nickel and iron oxyhydroxide electrocatalysts

Laia Francàs[1,4]*, Sacha Corby[1,4], Shababa Selim[1], Dongho Lee[2], Camilo A. Mesa[1], Robert Godin[1], Ernest Pastor[1], Ifan E.L. Stephens[3], Kyoung-Shin Choi[2], James R. Durrant[1]*

1. Department of Chemistry, Imperial College London, White City Campus, London W12 0BZ, United Kingdom
2. Department of Chemistry, University of Wisconsin-Madison, Madison, Wisconsin 53706, United States
3. Department of Materials, Imperial College London, South Kensington Campus, London SW7 2 AZ, United Kingdom
4. These authors contributed equally to this work

*Corresponding authors: lfrancas@ic.ac.uk, j.durrant@imperial.ac.uk

Supplementary Methods

Elemental Analysis

The elemental analyses of the electrocatalysts, both as prepared and after electrochemical tests, were carried out by dissolving the electrocatalyst films in 10 mL of 10 wt. % nitric acid (Fisher Chemical, TraceMetal Grade) and the amount of Fe and Ni in the resulting solutions quantified using inductively coupled plasma optical emission spectroscopy (ICP-OES). All disposable pipette tips, polypropylene tubes, and beakers were pre-rinsed with ~5 wt. % nitric acid, followed by rinsing with DI water and drying in air prior to use to remove any residual Fe. ICP standard solutions of Fe and Ni with various concentrations (10, 50, 100, 300, and 500 ppb) were used for calibration and 100 ppb yttrium was used as an internal standard to compensate for the signal drift among samples (Sigma Aldrich 43149 (Fe), 28944 (Ni), and 01357 (Y)). The atomic % of Fe in Ni(Fe)OOH and FeOOHNiOOH samples was determined using Eq. 1. The ICP-OES data is summarized in Supplementary Table 1.

Equation 1. $$\text{Fe atomic \%} = \frac{\text{mol of Fe}}{(\text{mol of Fe}+\text{mol of Ni})} \times 100$$

Electrocatalyst Activation Process

The anodic electrodeposition methods used in this study produced FeOOH and NiOOH films. While FeOOH is stable in the air, black NiOOH gradually changes to transparent $Ni(OH)_2$ in the air when it is not anodically protected. Therefore, Ni(Fe)OOH and FeOOHNiOOH samples were activated before optical and electrochemical experiments to convert $Ni(OH)_2$ in these samples to NiOOH. This process consists with the oxidation of the $Ni^{II}(OH)_2$ to the higher valence NiOOH, leading to strong absorbance assigned to metal-to-oxygen charge transfer transition. To do this, the potential was swept from the open circuit potential of the sample to 1.5 V vs. the Ag/AgCl RE (scan rate = 10 mV/s) five times. The conversion of $Ni(OH)_2$ to NiOOH could be confirmed visually by the return of the black color.

The percentage of Ni centres oxidised in the activation process for Ni(Fe)OOH and FeOOHNiOOH films was calculated from the spectroelectrochemistry data in Figure 2c in the main article and the ICP-OES results.

Ni(Fe)OOH → mol of Ni (SEC, Figure 2c) / mol of Ni (ICP, Sup. Table 1) = 3.9×10^{-8} / 4.63×10^{-8} = 0.85

➔ ~90%

FeOOHNiOOH → mol of Ni (SEC, Figure 2c) / mol of Ni (ICP, Sup. Table 1) = 5.7×10^{-9} / 6.22×10^{-8} = 0.09

➔ ~10%

Extinction Coefficient Estimation

Two different approaches have been used to estimate the extinction coefficient (Ɛ) of all the species.

(1) Ni(Fe)OOH(+): This extinction coefficient was estimated using the Lambert-Beer law combining linear sweep voltammetry experiments and optical measurements. We integrated the current under the redox wave in Supplementary Figure 4 to estimate the number of electrons involved in this process. The absorbance at 528 nm is taken from the UV-Vis spectrum presented in Supplementary Figure 5b.

Equation 2 $A = \varepsilon \times c$ where A = Absorbance at a particular wavelength, Ɛ is the extinction coefficient and c is the concentration of electrons per cm^2.

A (@528 nm) = 0.324
c = 2.362×10^{16} electrons/cm^2

$$\varepsilon = \frac{0.324}{2.362 \times 10^{16}} = 1.37\, x\, 10^{-17}\, \frac{cm^2}{number\ of\ e^-} \rightarrow 8250\, M^{-1}cm^{-1}$$

The obtained number in cm^2/number of e^- can be transformed to M^{-1} cm^{-1} as follows:

$$1.37 \times 10^{-17}\, \frac{cm^2}{No\ e^-} \times \frac{cm}{cm} \times \frac{6.022 \times 10^{23}\, no\ e^-}{1\ mol\ e^-} \times \frac{1\ dm^3}{1000\ cm^3} = 8250\, M^{-1}cm^{-1}$$

(2) FeOOH(++), FeOOHNiOOH(++) and Ni(Fe)OOH(++): The extinction coefficient of the second oxidized species in each case was estimated using the SP-SEC technique. We monitored the changes in absorbance when a voltage step was applied. The recorded optical data is proportional to the population of the oxidized states at the applied potential (Supplementary Figures 10a-12a). Simultaneously, the corresponding current is measured (Supplementary Figures 10b-12b) with an increase in current at the high applied potential and a reductive spike observed once the potential is switched to a lower one. The latter spike corresponds to the reduction of the accumulated oxidized states at the high potential region. The integration of these reductive currents (Inset Supplementary Figures 10b-12b) allow us to quantify the electrons used to reduce the oxidized states. Using the Lambert-Beer law (equation 2) we can estimate the corresponding extinction coefficients by plotting ΔO.D. against the electrons extracted (Supplementary Figures 10c-12c). The slope of the corresponding graphs yields the Ɛ in cm^2/number of e^-, which can be transformed to M^{-1} cm^{-1} as previously detailed.

Approximation of (+) and (++) quantities per sample

The quantity of [+] and [++] species can be calculated as a percentage of the total number of metal centres from the ICP-OES analysis given in Supplementary Table 1. The results are summarised in Supplementary Table 2. An alternative method of comparing the approximate (+) and (++) species concentrations in Ni(Fe)OOH is demonstrated in Supplementary Figure 20.

[+]

% FeOOH[+] → mol of Fe[+] (SEC, Figure 2c) / mol of Fe (ICP, Sup. Table 1) = $1.4 \times 10^{-9} / 2.4 \times 10^{-7} = 0.005$

➔ ~0.5%

% FeOOHNiOOH[+] → mol of Ni[+] (SEC, Figure 2c) / mol of Ni (ICP, Sup. Table 1) = $5.7 \times 10^{-9} / 6.22 \times 10^{-8} = 0.09$

➔ ~9%

% Ni(Fe)OOH[+] → mol of Ni[+] (SEC, Figure 2c) / mol of Ni (ICP, Sup. Table 1) = $3.9 \times 10^{-8} / 4.63 \times 10^{-8} = 0.85$

➔ ~85%

[++]

% FeOOH[++] → mol of Fe[++] (SEC, Figure 2c) / mol of Fe (ICP, Sup. Table 1) = $9.8 \times 10^{-9} / 2.4 \times 10^{-7} = 0.04$

➔ ~4%

% FeOOHNiOOH[++] → mol of Fe[++] (SEC, Figure 2c) / mol of Fe (ICP, Sup. Table 1) = $2.2 \times 10^{-8} / 2.4 \times 10^{-7} = 0.09$

➔ ~9%

% Ni(Fe)OOH[++] → mol of Ni[++] (SEC, Figure 2c) / mol of Ni (ICP, Sup. Table 1) = $5.3 \times 10^{-9} / 4.6 \times 10^{-8} = 0.12$

➔ ~12%

TOF (s^{-1}) and τ (s) calculation from measured [++] state densities and electrochemically measured water oxidation current densities.

In order to calculate these parameters we need the number of accumulated [++] (cm^{-2}), which come from the conversion of the obtained ΔO.D. using the extinction coefficient. On the other hand the current density data (A/cm^2) can be transformed into number of electrons/s using the electron charge (1.60×10^{-19} Coulombs).

For TOF calculation we consider that to produce one molecule of oxygen 4 oxidized species are needed

Equation 3. $TOF\ (s^{-1}) = \frac{number\ of\ electrons \times s^{-1}}{4\ [++]}$

τ is the lifetime of the oxidized species MOOH(++)

Equation 4. $\tau\ (s) = \frac{[++]}{number\ of\ electrons \times s^{-1}}$

Supplementary Tables

Supplementary Table 1. Summary of the ICP-OES results.

Samples	Fe content (10^{-8} mol/cm^2)	Ni content (10^{-8} mol/cm^2)	Fe at. %
FeOOH as prepared	23.50	0.10	100
Ni(Fe)OOH as prepared	0.05	4.63	1
Ni(Fe)OOH after investigation	0.25	4.38	5
FeOOHNiOOH as prepared	23.55	6.22	79
FeOOHNiOOH after investigation	23.76	6.24	79

Supplementary Table 2. (+) and (++) species per M

Samples	% [+] species per total M-centres*	% [++] species per total M-centres*
FeOOH	0.5	4
FeOOHNiOOH	9	9
Ni(Fe)OOH	85	12

*where 'M' is the active metal MOOH(++) species for each sample type.

Supplementary Figures

Sample Characterization

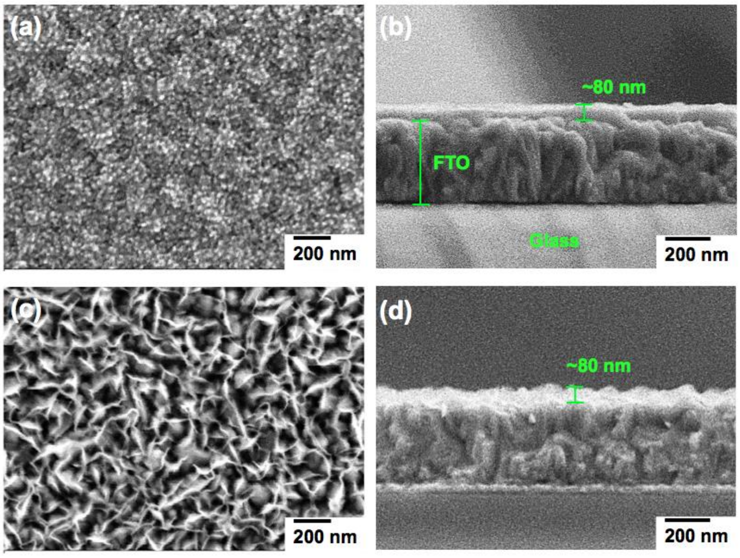

Supplementary Figure 1. SEM images of FeOOH and Ni(Fe)OOH: (a) Top view and (b) side view of FeOOH; (c) Top view and (d) side view of Ni(Fe)OOH.

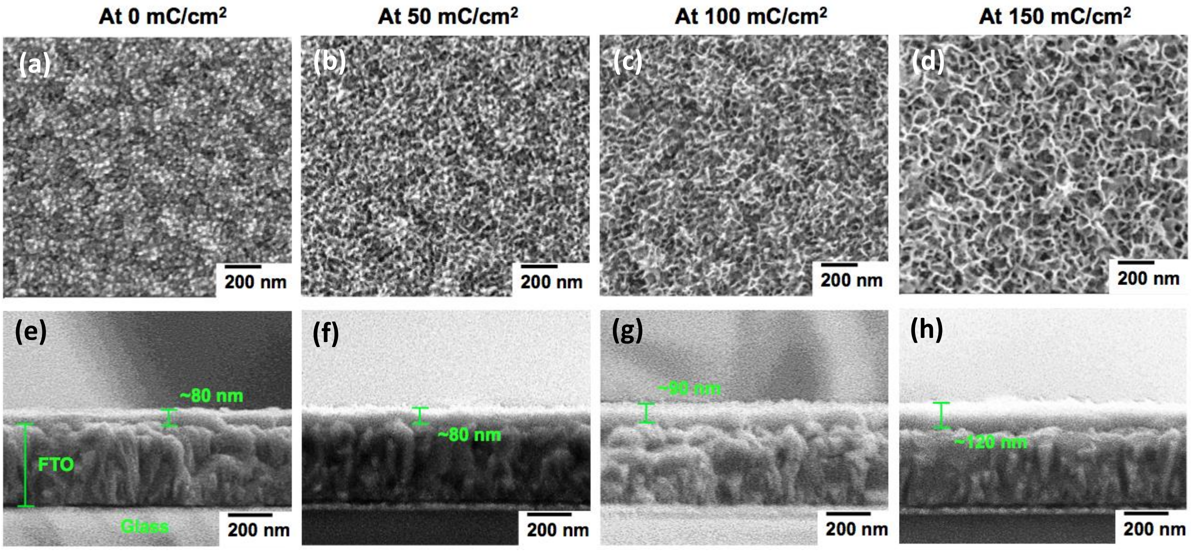

Supplementary Figure 2. SEM images for the deposition of NiOOH on top of pre-deposited FeOOH to form FeOOHNiOOH: Top view {(a)(b)(c)(d)} and side view {(e)(f)(g)(h)} images of FeOOHNiOOH films taken after passing (a)(e) 0 mC/cm^2, (b)(f) 50 mC/cm^2, (c)(g) 100 mC/cm^2, and (d)(h) 150 mC/cm^2 for the NiOOH deposition. The changes in the morphology and film thickness are not significant until passing 100 mC/cm^2, suggesting that up until passing 100 mC/cm^2, most NiOOH was deposited in the voids of the FeOOH layer resulting in significant mixing of Fe and Ni.

(a) (b)

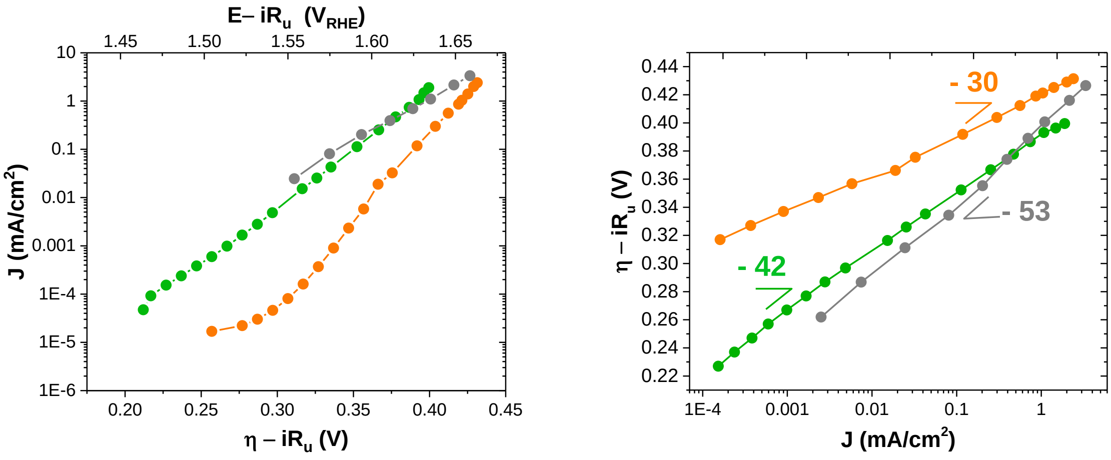

Supplementary Figure 3. Steady state current- voltage representations: (a) J-V curve with logarithmic scale for the current density; (b) Tafel plot with the different slopes indicated.

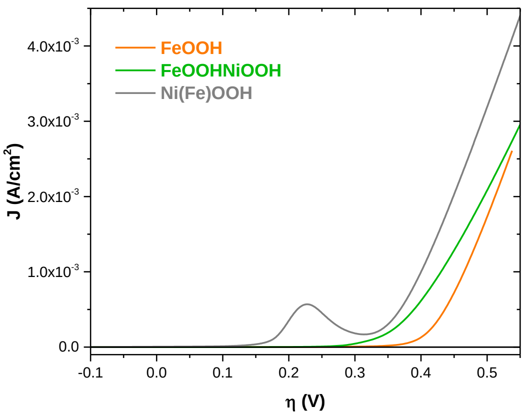

Supplementary Figure 4. The linear sweep voltammograms (LSVs) of Ni(Fe)OOH (gray) and FeOOHNiOOH (green) recorded during the first potential sweep of the activation process in 0.1 M NaOH. The LSV of FeOOH (orange) is also shown (scan rate = 10 mV/s). The distorted slope for the FeOOHNiOOH is not present on the activated sample as can be observed in Figure 1 in the main article.

Optical Measurements

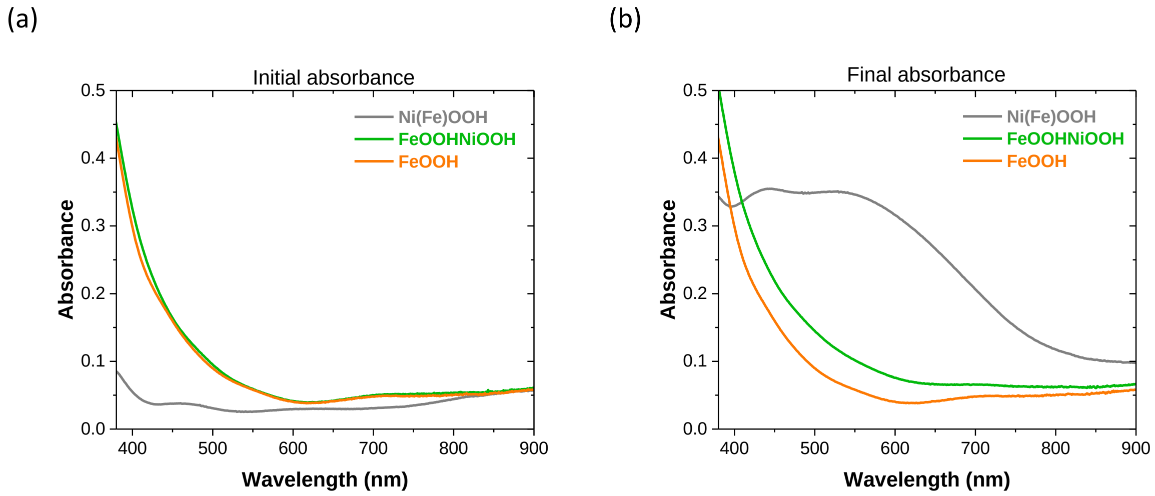

Supplementary Figure 5. UV-Vis absorbance spectra for FeOOH (orange), Ni(Fe)OOH (grey) and FeOOHNiOOH (green): (a) before activation; (b) after activation, see experimental section for further details.

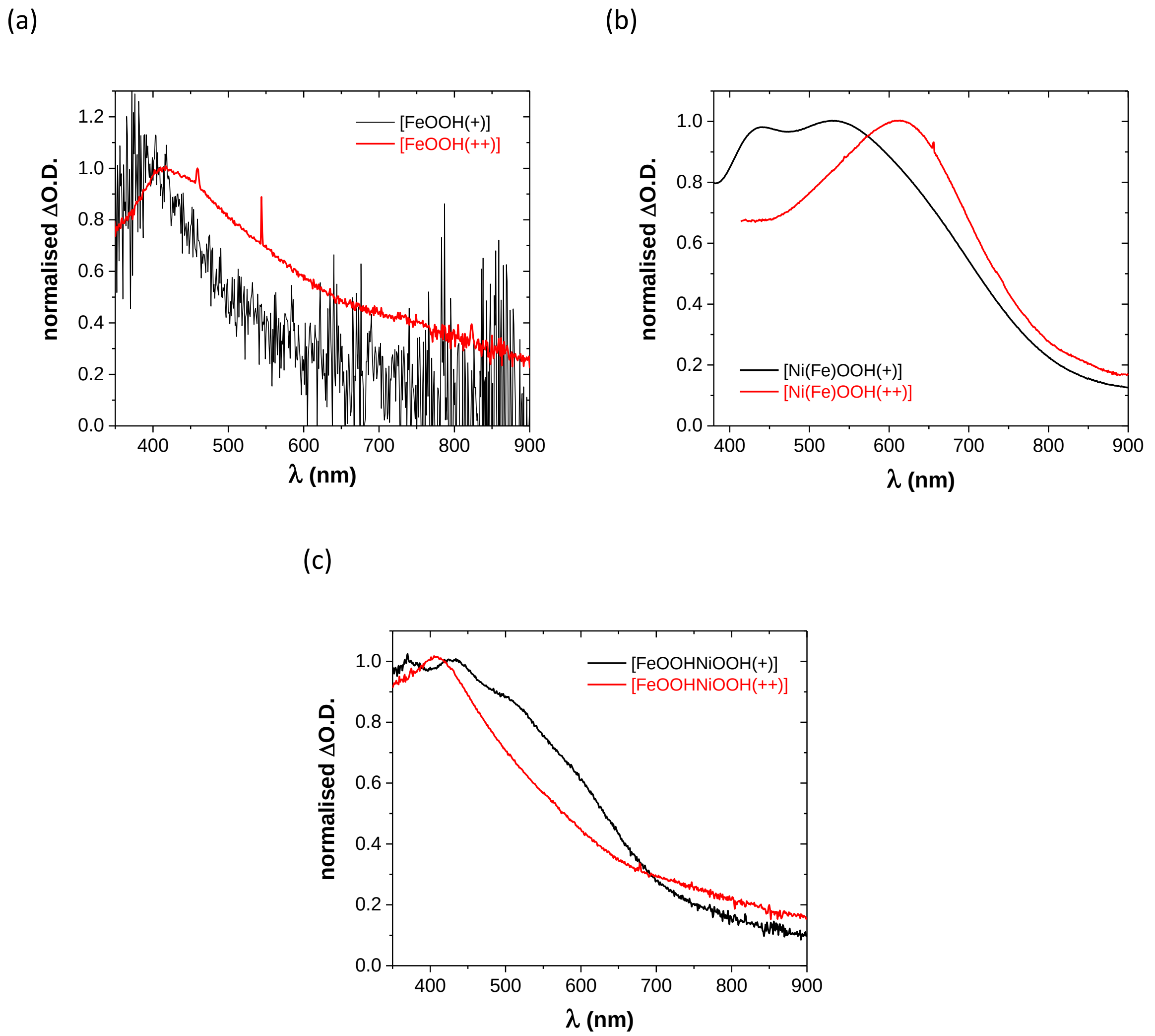

Supplementary Figure 6. Comparison between the normalized UV-Vis spectra of pre-catalytic (black) and the catalytic species (red) for: (a) FeOOH; (b) Ni(Fe)OOH and (c) FeOOHNiOOH. The change in absorption of the precatalytic species in Ni(Fe)OOH and FeOOHNiOOH was obtained by subtracting the initial absorbance from the absorbance after the activation. FeOOH precatalytic species spectrum was obtained by subtracting the absorbance at the open circuit potentials (0.237 V overpotential) from the absorbance at the onset of catalysis (0.307 V overpotential).

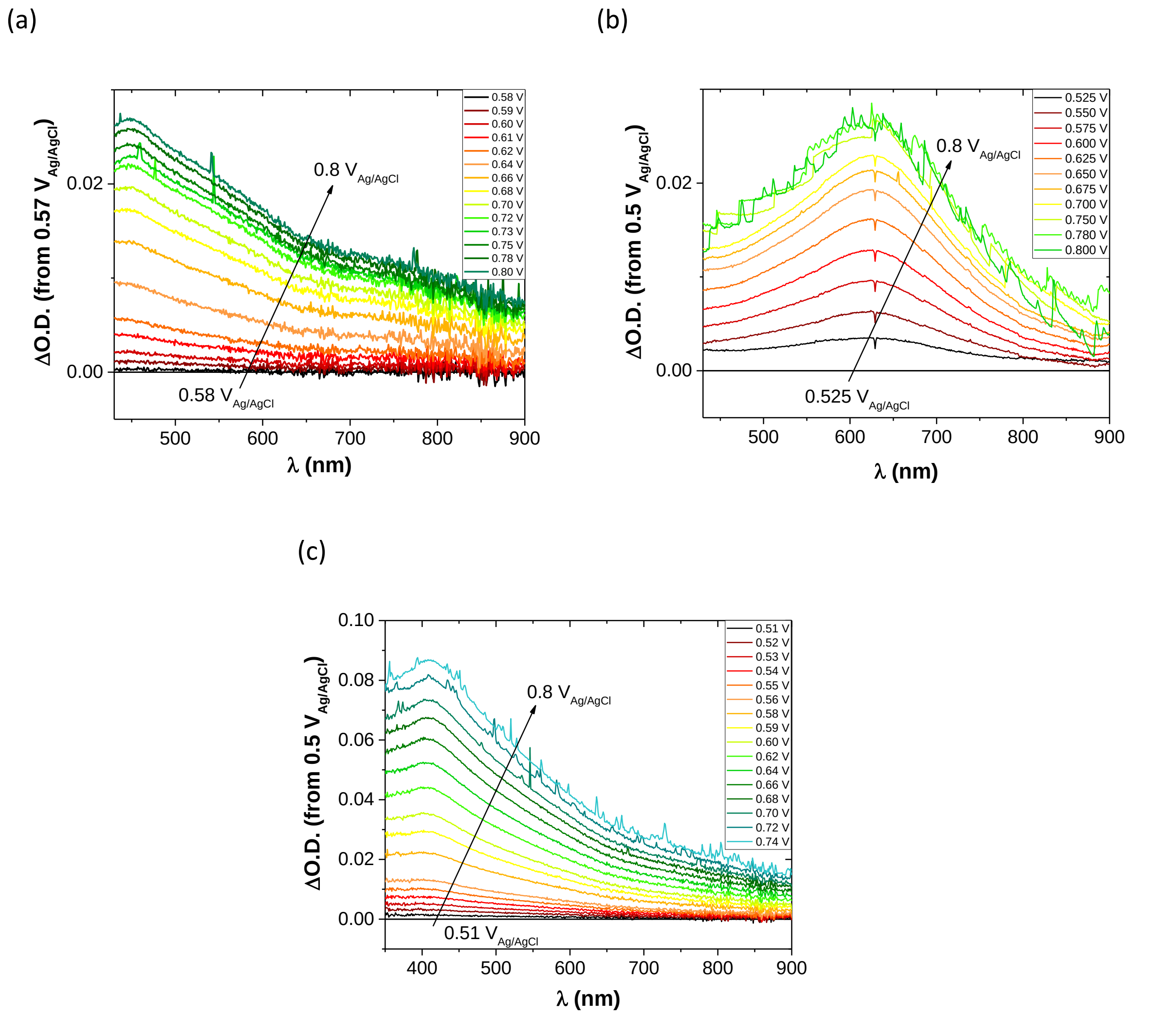

Supplementary Figure 7. UV-Vis spectra of the catalytic species at different applied potentials for (a) FeOOH, (b) Ni(Fe)OOH and (c) FeOOHNiOOH. The ΔO.D. was estimated by subtracting the absorbance at the catalytic onset from higher applied potentials within the catalytic region.

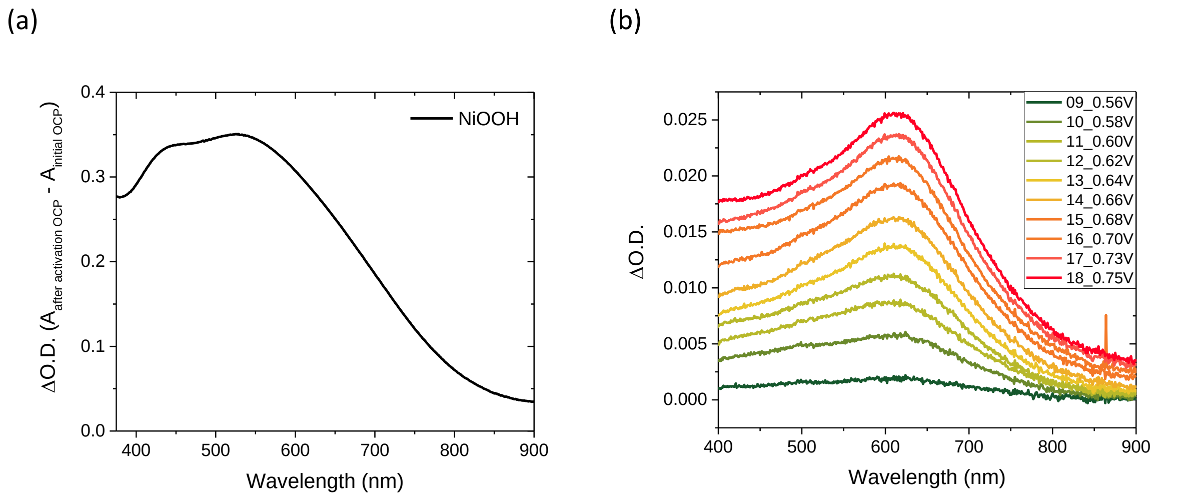

Supplementary Figure 8. (a) ΔO.D. spectra for the first (0/+), precatalytic, oxidation of NiOOH, obtained from the difference between the spectra at open circuit potential (OCP) before and after activation (as for Figure 2a in main article). (b) Difference spectra of the catalytic species at different applied potentials (given vs Ag/AgCl reference electrode) for NiOOH with no Fe impurities and measured in purified 0.1 M NaOH. As with the spectra given in Supplementary Figure 7, the ΔO.D. was estimated by subtracting the absorbance at the catalytic onset from higher applied potentials within the catalytic region. The peak clearly matches that found in Ni(Fe)OOH (Supplementary Figure 7b), thus confirming that the catalytic species is similar in nature and therefore Ni-centred.

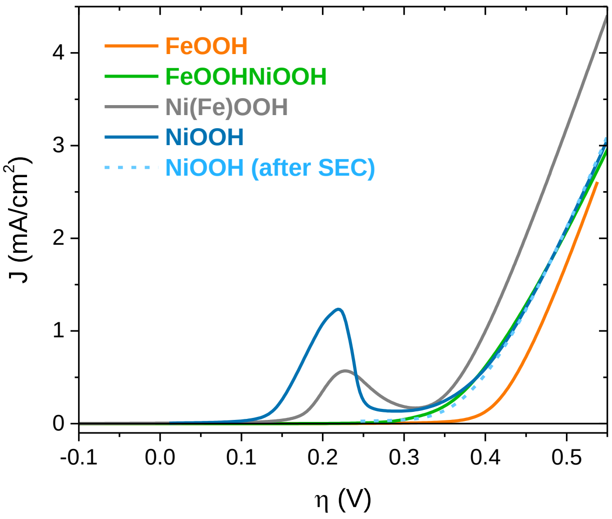

Supplementary Figure 9. The first linear sweep (LSV) of the pure NiOOH (dark blue) compared against the original electrocatalysts (Ni(Fe)OOH (grey), FeOOHNiOOH (green) and FeOOH (orange)) in 0.1 M NaOH. Despite the larger redox wave, suggesting the conversion of more $Ni(OH)_2$ to NiOOH, the catalytic current at 0.5 V overpotential is much worse than for Ni(Fe)OOH. The LSV after this sample was used for SEC is also provided (light blue) showing no change in performance, and thus confirming no Fe incorporation.

Extinction Coefficient Estimation

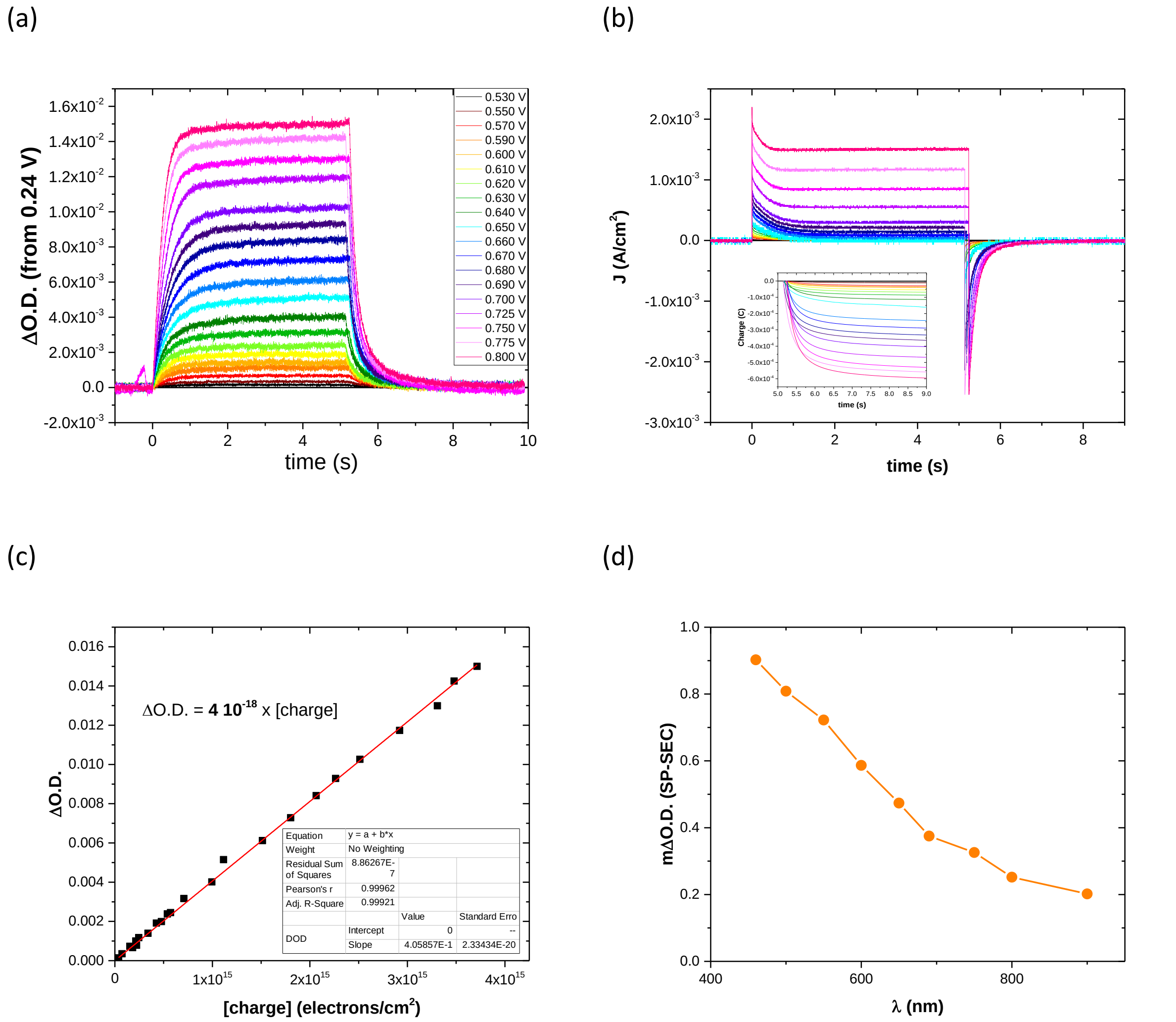

Supplementary Figure 10. SP-SEC for FeOOH from 0.24 V overpotential to 0.54 V (legend referenced against Ag/AgCl). (a) Optical data at 500 nm, (b) current density; inset, charge used to reduce the accumulated oxidised species and (c) optical signal shown versus the charge. Linear fit is shown as red line. (d) Spectra of the species generated at 0.5 V of overpotential using SP-SEC.

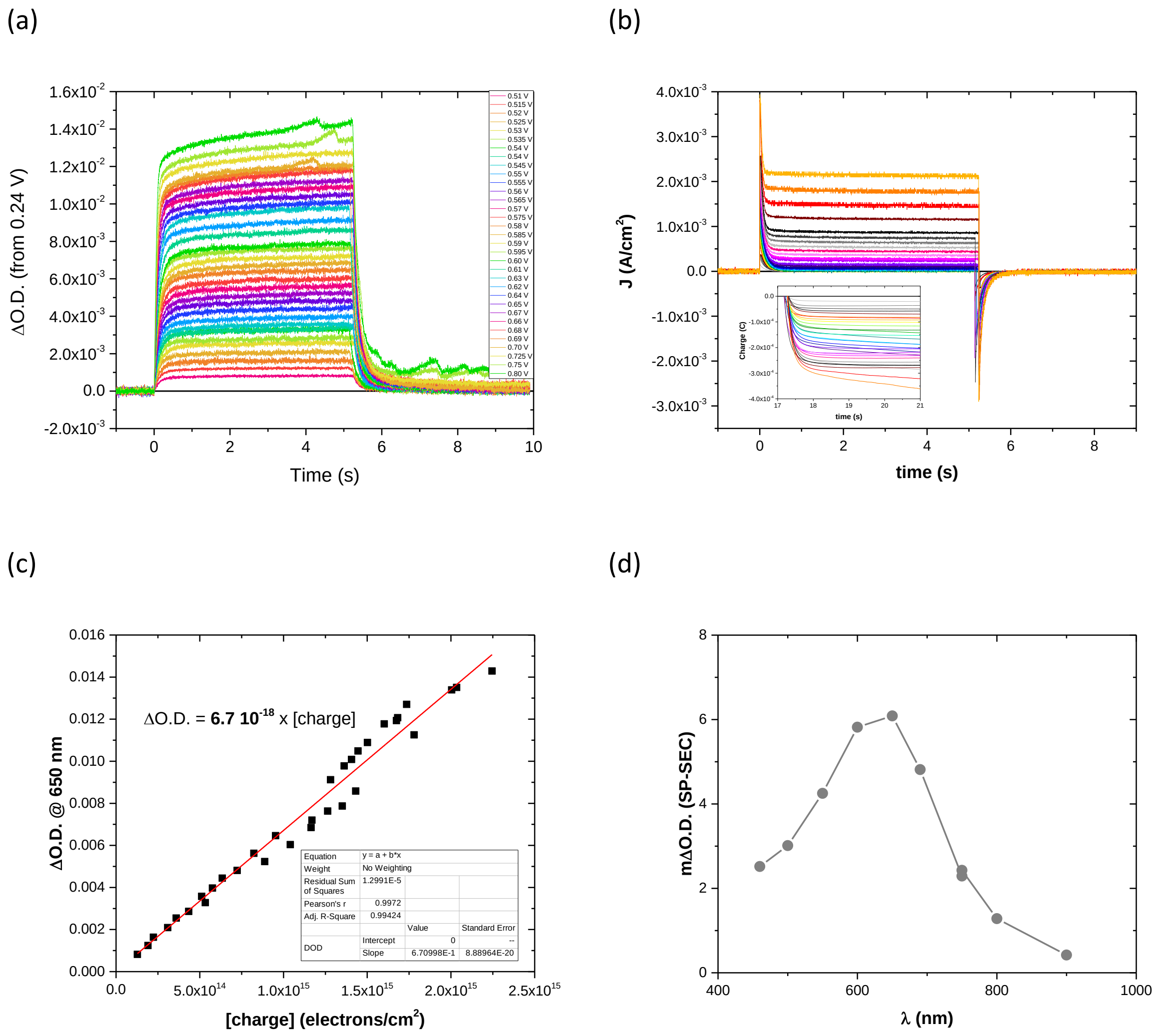

Supplementary Figure 11. SP-SEC for Ni(Fe)OOH from 0.24 V overpotential to 0.54 V (legend referenced against Ag/AgCl). (a) optical data at 650 nm, (b) Current density; inset, charge used to reduce the accumulated oxidised species and (c) optical signal shown versus the charge. Linear fit is shown as red line. (d) Spectra of the species generated at 0.5 V of overpotential using SP-SEC.

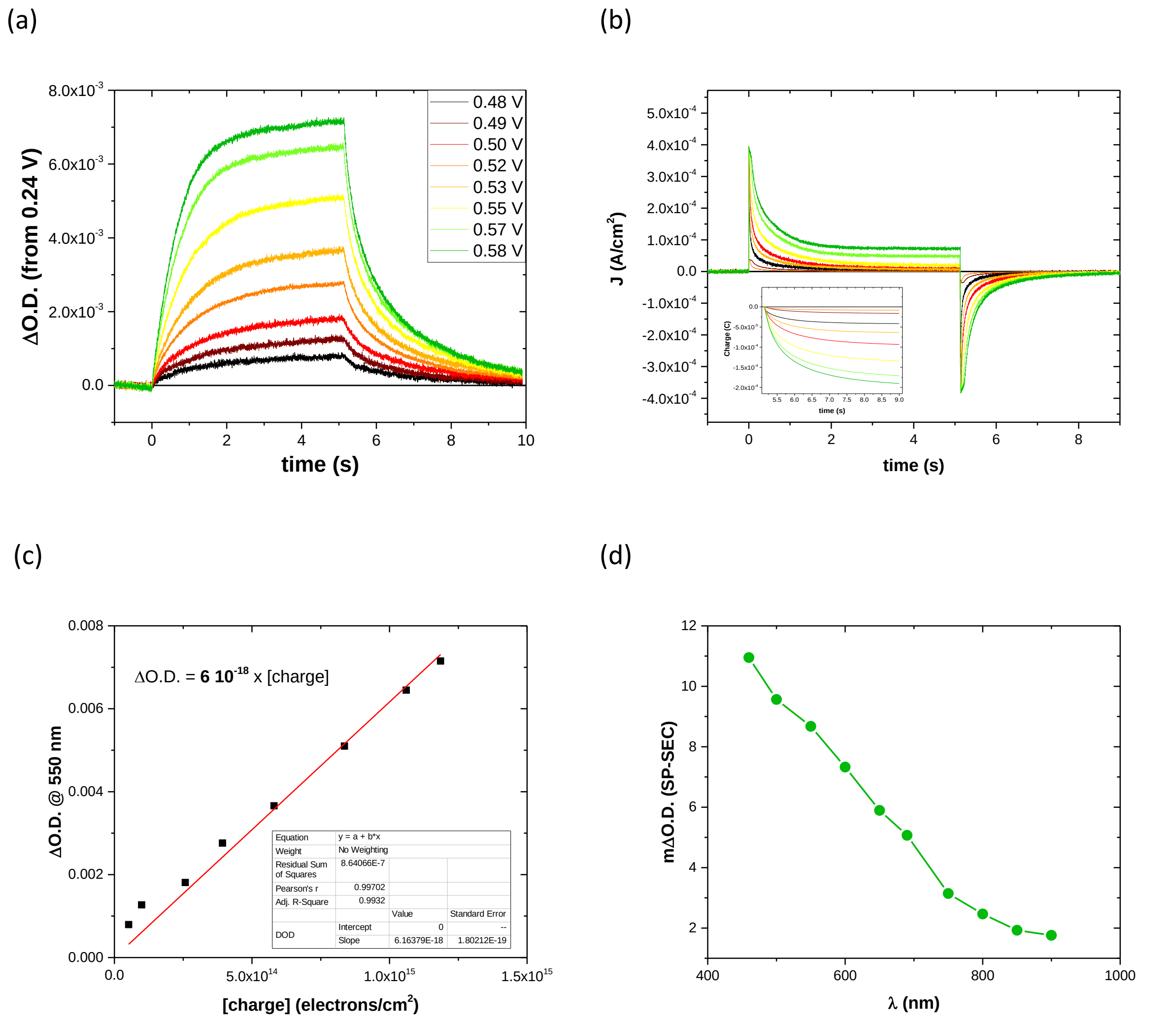

Supplementary Figure 12. Sp-SEC for FeOOHNiOOH from 0.24 V overpotential to 0.54 V (legend referenced against Ag/AgCl, same for (a) and (b)). (a) optical data at 550 nm, (b) Current density; inset, charge used to reduce the accumulated oxidised species and (c) optical signal shown versus the charge. Linear fit is shown as red line. (d) Spectra of the species generated at 0.5 V of overpotential using SP-SEC.

Optical Measurements in Organic Solvent

In order to determine if water is involved in the second oxidation of FeOOH, we carried out spectroelectrochemical experiments in propylene carbonate. As can be observed in Supplementary Figure 13c, in the absence of water the catalytic current is very low and shifted to higher potentials. The addition of 2.5% water induces an oxidative process seen around 1.2 V vs Ag/AgCl. This process can be tentatively assigned to the generation of the catalytic **FeOOH(++)** species, which cannot be generated in the absence of water. This is confirmed by spectroelectrochemical experiments in water (Supplementary Figure 13a) and in the absence of water (Supplementary Figure 13b). As shown, the change in absorbance, and thus the formation of the **(++)** species, is dependent on contact with water and does not form in its absence.

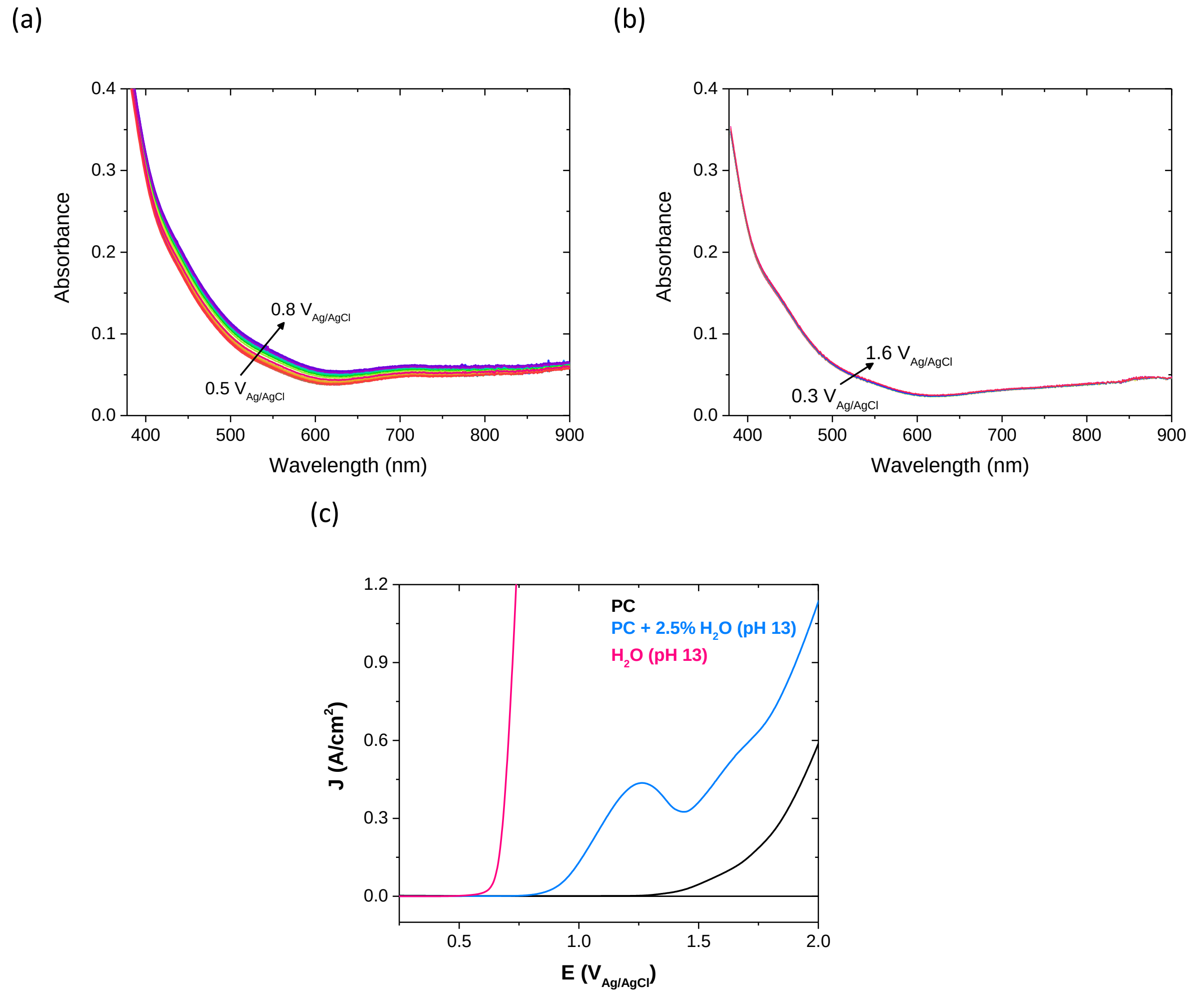

Supplementary Figure 13. Study of the aqueous solvent effect in FeOOH. (a) UV-Vis spectra of FeOOH at different applied potentials at pH 13. (b) UV-Vis spectra of FeOOH at different applied potentials in Propylene Carbonate (PC) 0.1 M NBu_4PF_6 solution. (c) J-V curves of FeOOH in pure PC 0.1 M NBu_4PF_6 (black), containing 2.5% of pH 13 aqueous solution (blue) and pure pH 13 aqueous solution (pink).

Relationship Between (0/+) and (+/++) Processes

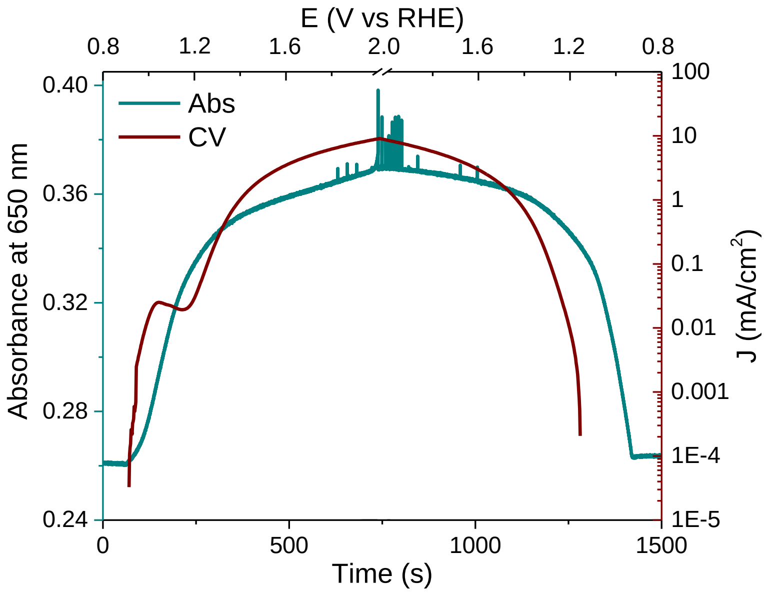

Supplementary Figure 14. Simultaneous measurement of the optical signal at 650 nm (teal) and current (burgundy) during CV cycling of a sample of Ni(Fe)OOH at 1 mV/s in 0.1 M NaOH. At the ca. the catalytic onset, the absorbance profile changes shape, reflecting a shift from the accumulation of [+] to [++] species.

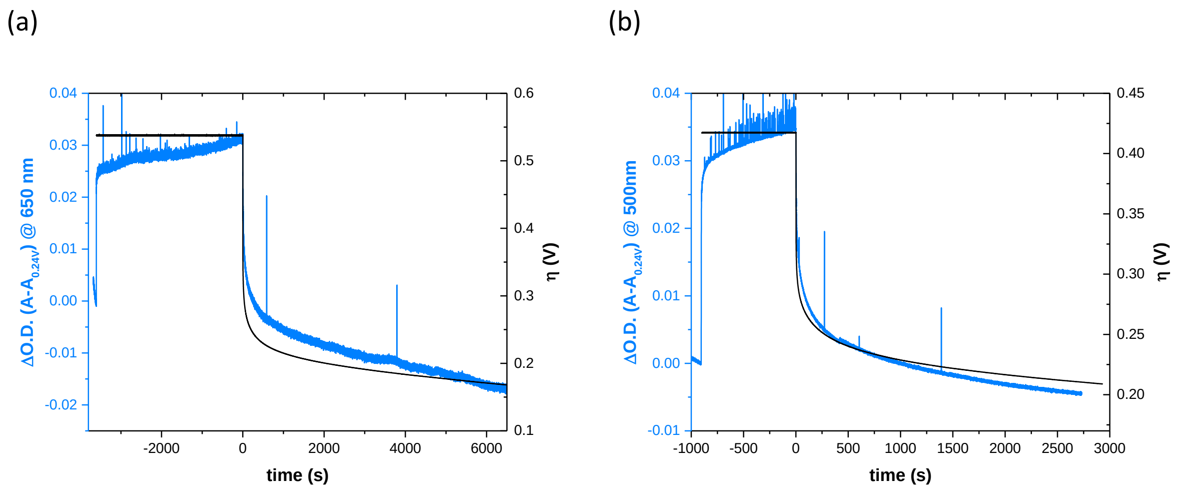

Supplementary Figure 15. Simultaneous measurement of the optical signal (blue trace) and applied potential (black trace) during catalytic current and after turning off the potential (~1.7 V vs RHE), from this moment the open circuit potential of the system was measured (black trace). (a) Ni(Fe)OOH and (b) FeOOHNiOOH.

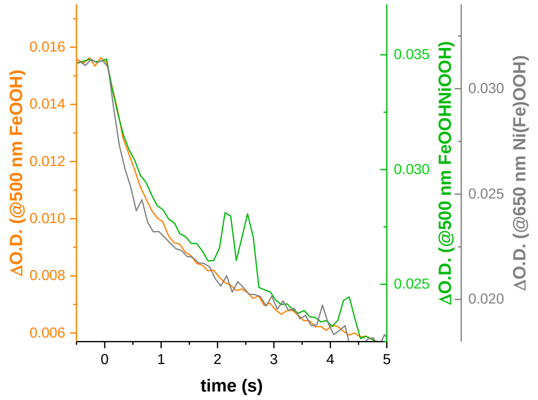

Supplementary Figure 16. Optical signal at a catalytic applied potential (~1.7 V vs RHE) and after turning off the potential. (Orange) FeOOH, (grey) Ni(Fe)OOH and (green) FeOOHNiOOH.

Rate Law and Reaction Order

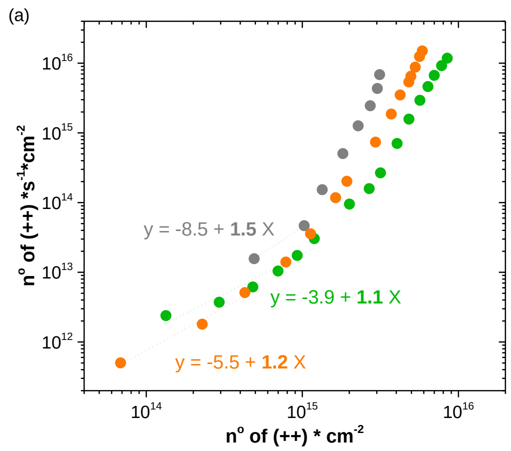

Supplementary Figure 17. Rate law plot at lower current densities for (orange) FeOOH, (grey) Ni(Fe)OOH and (green) FeOOHNiOOH.

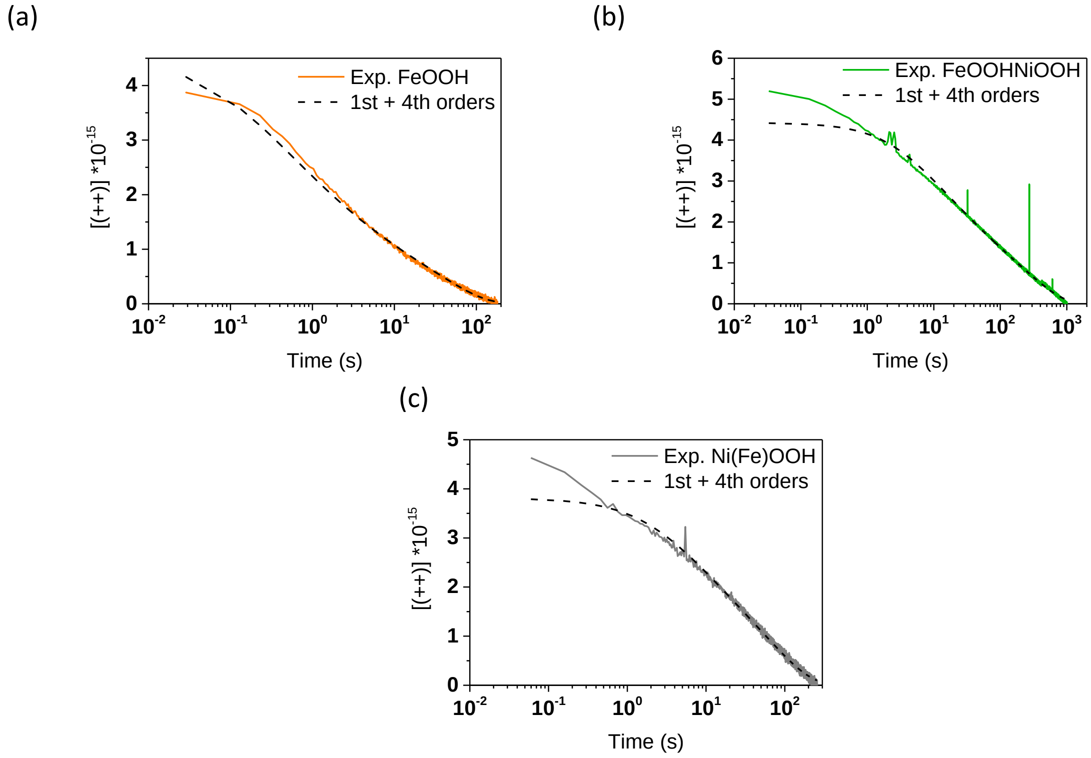

Supplementary Figure 18. Optical decay fitting for electrocatalyst when catalytic overpotential (~1.7 V vs RHE) has been turned off. (a) FeOOH (orange solid line) experimental decay; calculated decay (black dashed line) with the equation: d[FeOOH(++)]/dt = 1.88 x10^{-2} s^{-1}+ 1.88 x 10^{-47} cm^6 x no electrons^{-3} x s^{-1}
(b) FeOOHNiOOH (green solid line) experimental decay; calculated decay (black dashed line) with the equation: d[FeOOHNiOOH(++)]/dt = 2.75 x10^{-3} s^{-1}+ 7.63 x 10^{-49} cm^6 x no electrons^{-3} x s^{-1}
(c) Ni(Fe)OOH (grey solid line) experimental decay; calculated decay (black dashed line) with the equation: d[FeOOHNiOOH(++)]/dt = 1.14 x10^{-2} s^{-1}+ 1.57 x 10^{-48} cm^6 x no electrons^{-3} x s^{-1}.

We note that the kinetic analysis given in Figure 4 in the main article relies upon accurate calculation of the extinction coefficient for the MOOH[++] species such that their concentration may be determined from optical data. On the other hand, the analysis presented here in Supplementary Figures 15 to 18 does not require such calculations, and the tau values in these cases (presented in Table 1, entry 8) are obtained only by fitting the raw kinetic traces. As such, the agreement in the tau values obtained from these independent measurements increases confidence in our analyses.

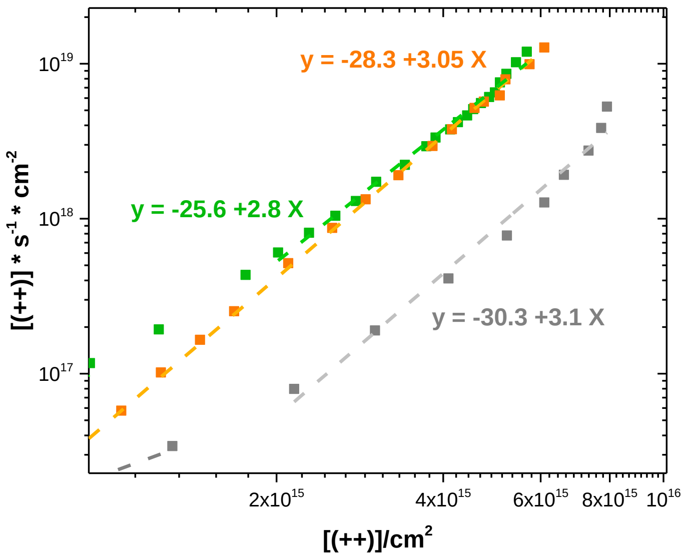

Supplementary Figure 19. Rate law plot for (orange) FeOOH, (grey) Ni(Fe)OOH and (green) FeOOHNiOOH at pH 7 (0.1 M K_2HPO_4/KH_2PO_4 buffer).

Approximation of (+) and (++) quantities per sample

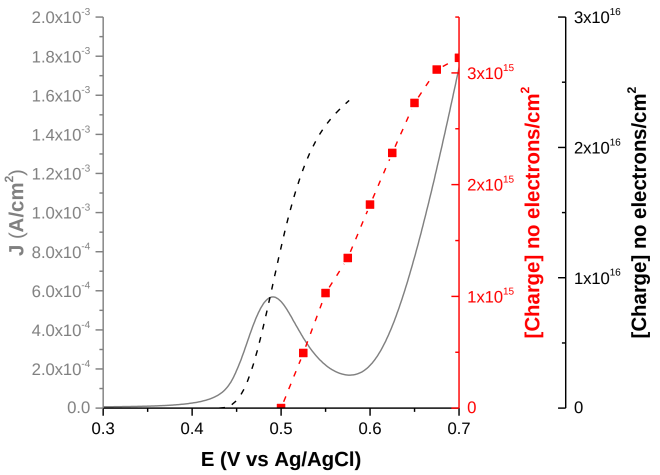

Supplementary Figure 20. Detailed study on the accumulated charge in Ni(Fe)OOH at pH 13. J-V curve before activation, where the oxidative wave from $Ni(OH)_2$ to NiOOH can be observed (solid grey line); Accumulated charge during the activation processes estimated by the integration of the charge below the $Ni(OH)_2$/NiOOH oxidative wave (black dashed line); Charge accumulated based on our optical measurements of the catalytic species (Red squares and dashed line).

Mechanistic Considerations

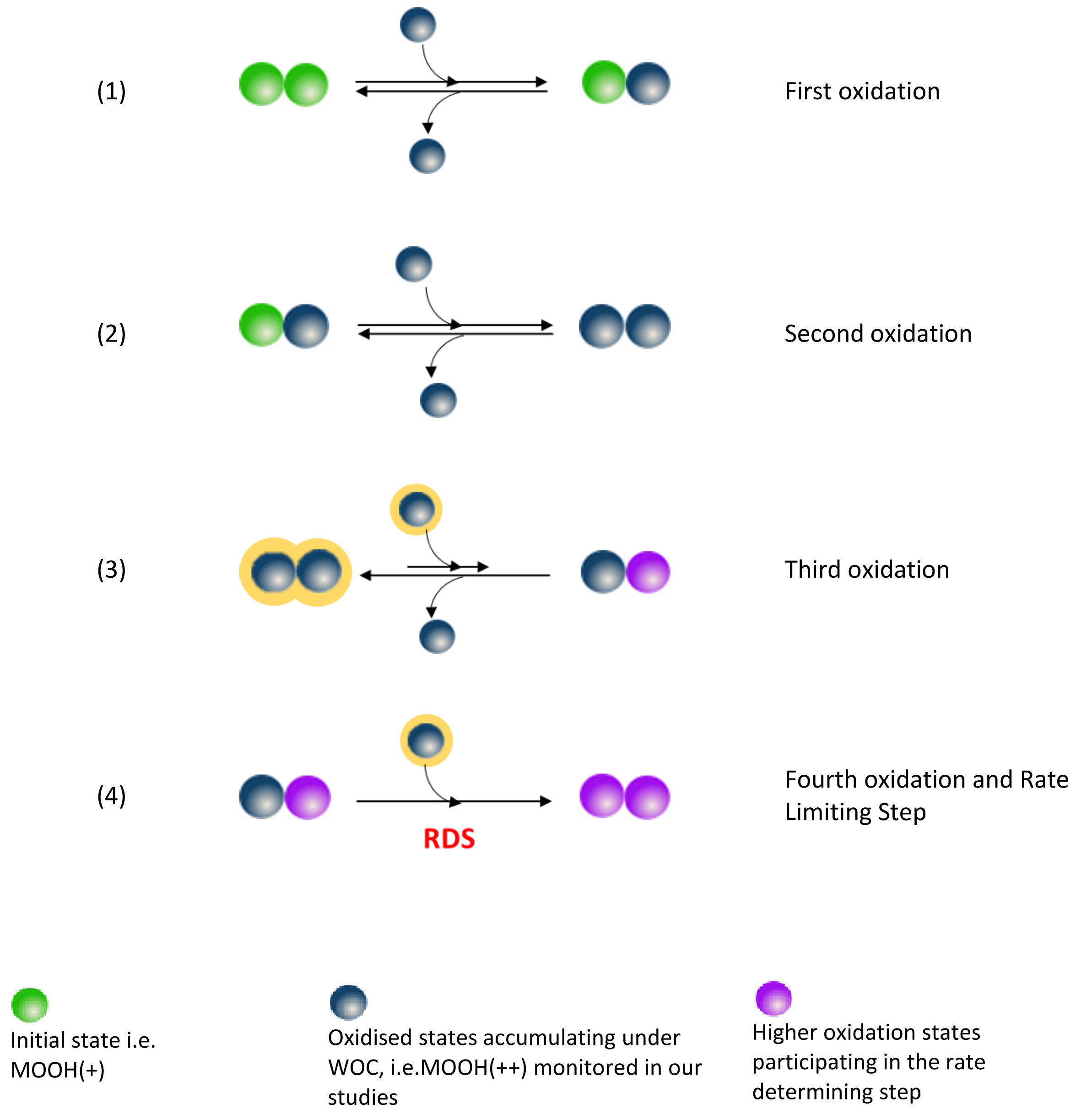

Supplementary Figure 21. Schematic representation of a possible order four mechanism considering a dimer as a reactive cluster. The spheres highlighted in yellow would be those observed under the steady state of the reaction. This merely acts as a visual aid to demonstrate how a 4th order reaction could arise in an example system.
